# Early newborn ritual foods correlate with delayed breastfeeding initiation in rural Bangladesh

**DOI:** 10.1186/s13006-016-0090-9

**Published:** 2016-12-08

**Authors:** Maria E. Sundaram, Hasmot Ali, Sucheta Mehra, Abu Ahmed Shamim, Barkat Ullah, Mahbubur Rashid, Saijuddin Shaikh, Parul Christian, Rolf D. W. Klemm, Keith P. West, Alain Labrique

**Affiliations:** 1 Department of Environmental Health, University of Minnesota School of Public Health, Minneapolis, MN USA; 2 The JiVitA Maternal and Child Health and Nutrition Research Project, Gaibandha, Bangladesh; 3 Center for Human Nutrition, Department of International Health, Johns Hopkins Bloomberg School of Public Health, Baltimore, MD USA; 4 SHIKHA Project, FHI 360, Durham, NC USA; 5 International Center for Diarrhoeal Disease Research, Dhaka, Bangladesh

**Keywords:** Breastfeeding, Neonatal health, Prelacteal, Early newborn food, South Asia

## Abstract

**Background:**

Early and exclusive breastfeeding may improve neonatal survival in low resource settings, but suboptimal breastfeeding still exists in areas with high infant mortality. Prelacteal feeding, the practice of giving a non-breastmilk food as a neonate’s first food, has been associated with suboptimal breastfeeding practices. We examined the association of feeding a non-breastmilk food in the first three days of life (early neonatal food, or ENF) with time from birth to initiation of breastfeeding among 25,286 Bangladeshi mother-neonate pairs, in a secondary analysis of a randomized controlled trial in northwestern rural Bangladesh conducted from 2001–2007.

**Methods:**

Trained interviewers assessed the demographic characteristics during pregnancy. At three months postpartum, the interviewers visited participants again and retrospectively assessed demographic and breastfeeding characteristics surrounding the birth. We assessed the relationship between ENF and time to initiation of breastfeeding in hours in both unadjusted and adjusted linear regression analyses. We also calculated reverse cumulative distribution curves for time to initiation of breastfeeding and analyses were stratified by an infant’s ability to breastfeed normally at birth.

**Results:**

The mean ± SD time from birth to initiation of breastfeeding was 30.6 ± 27.9 hours. Only 2,535 (10.0%) of women reported initiating breastfeeding in the first hour after birth and 10,207 (40.4%) reported initiating breastfeeding in the first 12 hours after birth. In adjusted linear regression analyses, feeding ENF was associated with a significant increase in time, in hours, to breastfeeding initiation both among children not able to breastfeed at birth (37.4; 95% CI 33.3, 41.5) and among children able to breastfeed at birth (13.3; 95% CI 12.7, 14.0).

**Conclusions:**

Feeding ENF was strongly associated with delayed initiation of breastfeeding, even after adjusting for other related factors and stratifying on the neonate’s ability to suckle normally after birth. More research is needed to understand the impact of these findings on optimal breastfeeding in this setting. It is possible that ENF feeding and the ability to breastfeed immediately after birth are interrelated in their respective associations to suboptimal breastfeeding initiation. This study in a large population representative of other populations in rural South Asia, demonstrates significantly longer times to breastfeeding initiation than previously appreciated, with a possible important role of ENF feeding.

**Trial registration:**

The randomized controlled trial on which this analysis is based, “Impact of Maternal Vitamin A or Beta-Carotene Supplementation on Maternal and Infant Mortality in Bangladesh”, was registered with ClinicalTrials.gov as trial number ID GHS-A-00-03-00019-00 and identifier NCT00198822. The identifier was first received September 12, 2005 (retrospectively registered). The first participant was enrolled in August 2001.

**Electronic supplementary material:**

The online version of this article (doi:10.1186/s13006-016-0090-9) contains supplementary material, which is available to authorized users.

## Background

Breastfeeding has many potential health benefits for infants in low and middle income countries, including reducing risk of infection [1–4], malnutrition and undernutrition [5], and mortality [3, 6, 7]. The World Health Organization (WHO) recommends exclusive breastfeeding (feeding only breast milk and, if necessary, medicine) as the standard practice for newborn infants until six months of age [8]. Early initiation of breastfeeding (within an hour of birth) is also recommended by WHO [9] and delayed initiation has been associated with increased risk of infant mortality [10–14]. However, despite exclusive breastfeeding advocacy efforts and evidence that breastfeeding improves infant outcomes in Bangladesh [15], early and exclusive breastfeeding remains low [16–18]. A comprehensive study found a 47.1% prevalence of early breastfeeding and 64.1% prevalence of exclusive breastfeeding until six months in 2011 in Bangladesh [16]. Additionally, early complementary feeding continues to occur [19, 20].

Cultural practices that encourage the early introduction of non-breastmilk foods, prelacteal feeding, have previously been suggested to be disruptive to the optimal timing of breastfeeding initiation [21–24] and to maintaining exclusive breastfeeding as per WHO recommendations [25, 26]. A unique opportunity to conduct a cross-sectional analysis determining whether early newborn feeding (feeding a non-breastmilk food within the first three days of life) is a risk factor for delaying breastfeeding initiation arose within the context of a large cohort of pregnant women and new mothers enrolled in a prospective randomized trial in northwestern rural Bangladesh [27]. We used a retrospective survey, conducted at three months postpartum, to capture all non-breastmilk foods given to neonates within the first three days of life, a period when most breastfeeding is also initiated. In this context, over 590 community based workers ensured postnatal follow up with mothers, allowing all breastfeeding and ENF data to be collected. In this analysis, we refer to these foods as “early neonatal foods” (ENF) because we are unable, through this survey, to establish with sufficient precision whether a food had been given before the initiation of breastfeeding. The objective of this analysis was to explore possible relationships between ENF and suboptimal breastfeeding initiation.

## Methods

### Participants and study design

We conducted an analysis of data collected for the JiVitA Project in rural Gaibandha, Bangladesh. The details of this randomized controlled trial, “Impact of Maternal Vitamin A or Beta-Carotene Supplementation on Maternal and Infant Mortality in Bangladesh”, have been published elsewhere [27, 28]. In brief, between 2001 and 2007, pregnant women were enrolled continuously into a cluster randomized, placebo and controlled community trial testing the effects of a weekly supplement of either vitamin A or beta-carotene on maternal mortality in northwestern rural Bangladesh. Each participant was interviewed during early pregnancy and at three months postpartum by trained, community based interviewers. Time to breastfeeding initiation was assessed by asking the mother “How many hours after birth did you start to breastfeed this child?” Feeding ENF was assessed by asking “Was anything other than mother’s breastmilk offered to the child within three days after birth?”

### Statistical analysis

We used linear regression models to assess the relationship between ENF feeding and the number of hours after birth that breastfeeding began. Potential confounders were identified *a priori* from factors identified in the breastfeeding literature and included independent variables describing a best guess gestational age of the neonate at birth (defined below), wealth index (defined on a continuous scale as previously described [29]), maternal and child vitality scores (created for this analysis and described below), child sex, maternal age, child size at birth (as perceived by the mother), primigravid status, maternal literacy, whether a Caesarean section was performed at the birth, the location of the birth, and the type of birth attendant present. Child vitality scores consisted of the presence of any of the following characteristics (perceived and reported by the mother) at birth: small infant size, taking more than 1 minute to start breathing or crying, crying weakly, not moving at all, being blue all over or having blue hands or feet, or having convulsions in the first seven days. Maternal vitality scores consisted of the presence of any of the following self-reported symptoms in the seven days after giving birth: high fever, convulsions, vomiting, or dysentery. Each score was constructed by giving a point to each of the conditions listed above, so that higher scores reflect higher risk health states. Most often, the estimate of gestational age was informed by a precise date of the mother’s last menstrual period, derived from continuous longitudinal pregnancy surveillance and optimized by other available information such as positive urine testing [28]. Regression analyses were stratified by the maternal perception of her child’s ability to suckle normally at birth. The beta-coefficients of regression analyses are interpreted as additional hours to breastfeeding from the average. A beta-coefficient value above 0 indicates a delay in breastfeeding as compared to the average.

Diagnostics were performed to test for collinearity among variables in regression models. Participants were dropped from the analysis if they were missing information for any of the independent variables in the regression analysis. Additionally, we created reverse cumulative distribution curves to describe the time to initiation of breastfeeding by ENF feeding, using the Stata [distplot] module.

A value of *p* < 0.05 was considered significant. Stata 11 and Stata 14 (College Station, TX) were used for statistical analysis.

## Results

**Fig. 1:**
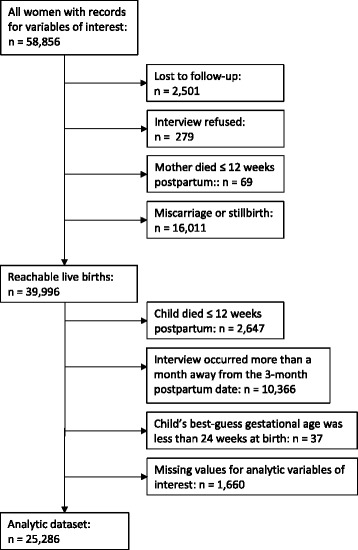
Flow chart of individuals included and excluded from the current analysis

There were 37,349 women with live births and children that survived past 12 weeks postpartum. After excluding mother-neonate pairs where the postpartum interview was conducted more than 28 days earlier or 28 days later than the three month postpartum mark (*n* = 10,366), premature births when women whose children had a best guess gestational age of less than 24 weeks at birth (*n* = 37), and women missing variables of interest (*n* = 1,660), a total of 25,286 women were included in the analysis (Fig. 1). Women who were excluded due to a late or early interview were similar to women included with regard to all analytic variables (maternal age, primigravid status, wealth index, maternal literacy, child sex, child size at birth, whether an early newborn food was fed, whether colostrum was fed, whether the child had difficulty suckling at birth, the location of birth, whether the child was delivered by Caesarean section, and the birth attendant type (Additional file 1). Women who were excluded took longer, on average, to begin breastfeeding (39.9 ± 28.2 hours to breastfeeding, compared to 30.6 ± 27.9 hours for women included). The majority of women (*n* = 22,650, 89.6%) fed their newborn an ENF, and 24,073 women (95.2%) fed colostrum (Table 1). The mean ± SD time to breastfeeding initiation from birth was 30.6 ± 27.9 hours (Table 1); the median (IQR) time to initiation was 24 (6–51) hours. Only 2,535 (10.0%) of women reported initiating breastfeeding in the first hour after birth; 10,207 (40.4%) reported initiating breastfeeding in the first 12 hours after birth. Among mothers feeding ENF, animal milk, honey, and sweetened water were the three most common foods. This was true both for babies able to suckle (34.3% fed milk, 26.2% fed honey, and 25.2% fed sweetened water) and not able to suckle (44.3% fed milk, 19.4% fed honey, and 22.5% fed sweetened water).

**Table 1:** Descriptive characteristics of neonates and mothers in the study area (*n* = 25,286)

Characteristic	Total: *n* = 25,286
Maternal age, years: mean ± SD	21.9 ± 5.7
Primigravid: *n* (%)	9,493 (37.5)
Wealth index: mean ± SD	0.01 ± 1.0
Maternal literacy: *n* (%)	12,002 (47.5)
Female child gender: *n* (%)	12,574 (49.7)
Perceived child size at birth^a^: *n* (%)	
Small	6,071 (24.0)
Normal	9,449 (37.4)
Large	9,766 (38.6)
Fed early newborn food (ENF) *n* (%)	22,648 (89.6)
Colostrum fed: *n* (%)	24,071 (95.2)
Difficulty suckling at birth: *n* (%)	7,466 (29.5)
Hours to breastfeeding initiation: mean ± SD	30.6 ± 27.9
Location of birth	
At home	23,680 (93.7)
At a family welfare visitor’s house	277 (1.1)
Clinic/hospital	670 (2.7)
Enroute, other, or don’t know	652 (2.6)
Baby delivered by Caesarean section	457 (1.8)
Birth attendant type: *n* (%)	
No one present	389 (1.5)
Friend/neighbor/relative	18,199 (72.0)
Traditional birth attendant	5,716 (22.6)
Health care professional	970 (3.8)
Other	4 (<0.1)

^a^Perceived by mother

**Fig. 2:**
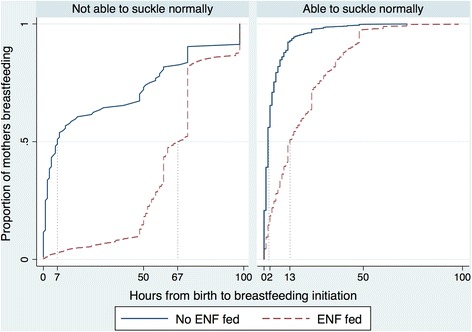
Reverse cumulative distribution curves illustrating time to breastfeeding and proportion of mothers feeding at that time, by neonate’s ability to breastfeed and feeding early newborn foods (ENF)

Reverse cumulative distribution curves revealed an increased average time to breastfeeding for all neonates when receiving ENF (Fig. 2). For neonates able to suckle normally at birth, those receiving ENF had a median time to breastfeeding initiation of 13 hours (compared to two hours for those not receiving ENF); for those unable to suckle normally at birth, those receiving ENF had a median time to breastfeeding of 67 hours (compared to seven hours for those not receiving ENF) (Fig. 2). ENF was associated with later initiation of breastfeeding in unadjusted models both among infants not able (36.47 additional hours to breastfeeding initiation; 95% CI 32.40, 40.54) and infants able to breastfeed at birth (14.07 additional hours to breastfeeding initiation; 95% CI 13.43, 14.70) (Table 2). In adjusted models controlling for best guess gestational age, wealth index, maternal and child vitality scores, child sex, maternal age, child size at birth (as perceived by the mother), primigravid status, maternal literacy, whether a Caesarean section was performed at the birth, the location of the birth, and the type of birth attendant present, ENF feeding was still significantly associated with increased time to breastfeeding initiation, both among infants not able (37.36 additional hours to breastfeeding initiation; 95% CI 33.26, 41.46) and infants able to breastfeed normally at birth (13.33 additional hours to breastfeeding initiation; 95% CI 12.69, 13.97) (Table 2).

**Table 2:** Risk factors associated with time to breastfeeding initiation (in hours) in univariable and multivariable linear regression analysis, stratified by ability to breastfeed at birth^a^

Characteristic	β (hours to breastfeeding initiation) (unadjusted)	95% CI	β (hours to breastfeeding initiation) (adjusted)^b^	95% CI
Not able to breastfeed at birth				
Early newborn food fed	36.47	32.40, 40.54	37.36	33.26, 41.46
Able to breastfeed at birth				
Early newborn food fed	14.07	13.43, 14.70	13.33	12.69, 13.97

^a^Coefficients described in the table are interpreted as additional hours to breastfeeding from the average. Numbers above 0 indicate a delay in breastfeeding compared to the average

^b^Adjusted model adjusts for best-guess gestational age, wealth index, maternal and child vitality scores, child sex, maternal age, child size at birth (as perceived by the mother), primigravid status, maternal literacy, whether a C-section was performed at the birth, the location of the birth, and the type of birth attendant present

## Discussion

The timing of initiation of breastfeeding within this population was suboptimal, with a mean delay of 30.6 hours between birth and initiation of breastfeeding. Women who fed an ENF initiated breastfeeding significantly later than women who did not feed ENF, even after controlling for other factors likely to hinder breastfeeding initiation and stratifying on the infant’s ability to breastfeed at birth. These results have been shown elsewhere: a recent meta-analysis indicated that early initiation of breastfeeding is low globally [30], and an additional systematic review identified prelacteal feeding as a major barrier to early initiation of breastfeeding in South Asia [31]. Other studies have shown a delay in breastfeeding associated with prelacteal feeding in Ethiopia [23, 32] and western Nepal [33].

In this population, which is demographically representative of rural Bangladesh and similar populations across the greater Gangetic floodplain [27, 28], early neonatal feeding is a common practice [20]. Mothers feeding ENF, and neonates’ inability to breastfeed after birth, are both associated with suboptimal breastfeeding practices. These factors may be interrelated in their respective associations with delayed breastfeeding initiation. For example, neonates who have trouble breastfeeding after birth or who are perceived to be too weak to breastfeed may be given a non-breastmilk food.

This study has at least four limitations. First, the information used in this analysis was derived from interviews performed both during pregnancy and at three months postpartum, and there is a possibility of recall bias. A three month recall bias has been shown to exist in reporting of breastfeeding behaviors [34]. However, this study focused on sensitivity and specificity of self-reported exclusive breastfeeding. The study results indicate that recall bias may overestimate optimal breastfeeding behaviors. Such a bias would reinforce the findings of this study. However, the interviews contained closed ended questions and were conducted by trained interviewers. To limit the possibility of recall bias, we excluded interviews carried out more than 28 days after or fewer than 28 days before the scheduled three month postpartum visit. Excluding interviews where the interview dates were closer to the dates of birth may have removed a subsection of interviews less subject to recall bias, however, this was done in the interest of maintaining internal validity. Women with interview dates similar to each other may be the most likely to give internally consistent interviews. There is a possibility that there may be differential misclassification of the exposure of interest (ENF feeding), based on recall bias about infant difficulty suckling (i.e., women who recall more difficulty suckling also are more apt to remember feeding ENF). However, we believe this possibility to be small due to the high prevalence of ENF feeding throughout the study population. Second, due to the timing of the study interview and the fact that mothers were not asked why they fed ENF, it is impossible to establish the direction of causality in the relationship between delayed initiation of breastfeeding and ENF feeding. Third, the participant interviews used in this analysis were conducted from 2001–2007, and may not describe more recent trends in early newborn feeding and early initiation of breastfeeding.

Finally, given the very large sample sizes of this analysis, we are powered to detect even small differences in the time to initiation of breastfeeding, including those that may be less clinically relevant. However, because of the ubiquity of ENF behaviors across populations in South Asia, rarely have studies been able to compare the attributes of ENF feeding with a very large group of individuals who did not provide their newborn ENFs. This statistical power provides a unique opportunity to identify both major and minor risk factors for delayed initiation of breastfeeding in this context, and allows us to be more confident about the lack of type II error in our statistical analysis.

## Conclusions

Our results indicate that ENF and neonatal difficulty of breastfeeding normally are both associated with delayed initiation of breastfeeding. Further research is needed to describe the causal relationship between ENF and delayed breastfeeding; the association shown does not establish whether ENF causes delayed breastfeeding, or whether delayed breastfeeding results in ENF being fed. A closely monitored prospective study may be better able to quantify the causal direction of this relationship. Further research is also needed to better characterize whether ENFs have consequences on neonatal health and survival, and whether exposure to ENF may introduce or expose vulnerable neonates to harmful pathogens or toxins. Any future research on early newborn feeding will be strengthened by a description of the sociocultural context in which ENF occurs for that particular study. It is, however, clear that this important association deserves careful scrutiny before ENFs are discounted as ‘harmless’ traditional practices.

## Additional file

Additional file 1: Table S1. Characteristics of women included and excluded in this analysis. (DOCX 91 kb)

## Abbreviations

ENF: Early newborn feeding
WHO: World Health Organization
