# Supplementary material for: Early newborn ritual foods correlate with delayed breastfeeding initiation in rural Bangladesh
Source: Int Breastfeed J. 2016 Dec 8;11:31. doi: 10.1186/s13006-016-0090-9 (PMC5143457; doi:10.1186/s13006-016-0090-9)
Supplement: Additional file 1: Table S1. — Characteristics of women included and excluded in this analysis. (DOCX 91 kb) [file 13006_2016_90_MOESM1_ESM.docx]

**Supplementary Table 1**. Characteristics of women included and excluded in this analysis.

| Characteristic | Not excluded (n = 25,286) | Excluded (n = 9,809)^a^ |
| --- | --- | --- |
| Maternal age, years: mean ± SD | 21.9 ± 5.7 | 21.7 ± 5.7 |
| Primigravid: N (%) | 9,493 (37.5) | 3,262 (33.3) |
| Wealth index: mean ± SD | 0.01 ± 1.0 | -0.1 ± 1.0 |
| Maternal literacy: N (%) | 12,003 (47.5) | 4,076 (41.6) |
| Female child gender: N (%) | 12,577 (49.7) | 4,775 (48.7) |
| Perceived child size at birth: N (%) |  |  |
| Small | 6,071 (24.0) | 2,409 (24.6) |
| Normal | 9,451 (37.4) | 3,325 (33.9) |
| Large | 9,766 (38.6) | 4,075 (41.5) |
| Early newborn food fed: N (%) | 22,650 (89.6) | 9,054 (92.3) |
| Colostrum fed: N (%) | 24,073 (95.2) | 9,192 (93.7) |
| Difficulty suckling at birth: N (%) | 7,467 (29.5) | 3,852 (39.3) |
| Hours to breastfeeding initiation: mean ± SD | 30.6 ± 27.9 | 39.9 ± 28.2 |
| Location of birth |  |  |
| At home | 23,682 (93.7) | 9,284 (94.7) |
| At a family welfare visitor’s house | 277 (1.1) | 69 (0.7) |
| Clinic/hospital | 670 (2.7) | 271 (2.8) |
| En route, other, or don’t know | 652 (2.6) | 184 (1.9) |
| Baby delivered by Caesarean section | 457 (1.8) | 156 (1.6) |
| Birth attendant type: N (%) |  |  |
| No one present | 389 (1.5) | 265 (2.7) |
| Friend/neighbor/relative | 18,200 (72.0) | 8,045 (82.0) |
| Traditional birth attendant | 5,717 (22.6) | 1,143 (11.7) |
| Health care professional | 970 (3.8) | 351 (3.6) |
| Other | 4 (< 0.1) | 2 (0.0) |

^a^ The manuscript states that 10,366 individuals were excluded due to an interview date more than a month away from the 3-month postpartum date. From this group, an additional 38 observations were excluded because of a best-guess gestational age less than 24 months at birth and 519 were missing values of interest for analysis.
